# Mapping the propagation of a salicylic acid wave during pathogen spread using a fluorescent sensor

**DOI:** 10.1016/j.abiote.2026.100055

**Published:** 2026-05-19

**Authors:** Shanshan Li, Honghong Cao, Qiuyue Zhang, Yukang Wang, Shancen Zhao, Ronghui Pan

**Affiliations:** a BLSA-ZJU Research Center, Zhejiang University, Hangzhou, 310058, China; b Zhejiang Key Laboratory of Intelligent Manufacturing for Functional Chemicals, ZJU-Hangzhou Global Scientific and Technological Innovation Center, Zhejiang University, Hangzhou, 310058, China; c State Key Laboratory of Rice Biology and Breeding, College of Agriculture and Biotechnology, Zhejiang University, Hangzhou, 310058, China; d Beijing Life Science Academy, Beijing, 102209, China

**Keywords:** Salicylic acid, Systemic acquired resistance, Biosensor, Real-time imaging, Plant immunity

## Abstract

Salicylic acid (SA) is a central signaling molecule in systemic acquired resistance (SAR) in plants, yet long-term monitoring of its real-time dynamics in living tissues has remained technically challenging. Using the recently developed genetically encoded fluorescent sensor SalicS1, high-resolution spatiotemporal imaging of SA was successfully performed in intact plants for the first time, revealing an SA wave that propagates ahead of the invading pathogen. This breakthrough tool directly captures the dynamic spread of plant defense signals. In addition, SalicS1 provides a technological foundation for dissecting the temporal relationships between SA and upstream early signals such as calcium ions and reactive oxygen species and for identifying novel immune signaling components through genetic screening. The development of this sensor marks a transition in plant immunity research from static measurements to real-time dynamic imaging.

Salicylic acid (SA) is an indispensable signaling molecule that functions in plant defense against pathogen invasion [1]. By orchestrating the initial triggering and systemic transmission of immune signals, SA activates downstream pathways, leading to the establishment of systemic acquired resistance (SAR). SAR is not akin to simply donning armor; instead, it activates a pervasive, dynamic chemical defense system throughout the entire plant [2]. After encountering localized pathogen attack, the plant rapidly enters a state of high alert, defending itself against subsequent widespread invasions. SA biosynthesis in plants occurs via two major pathways, the isochorismate synthase (ICS) pathway and the phenylalanine ammonia-lyase (PAL) pathway; the detailed pathways depend on plant phylogeny. In the *Brassicaceae* family, SA biosynthesis primarily relies on the ICS pathway. For example, Arabidopsis (*Arabidopsis thaliana*) exhibits a low basal SA level, which rapidly increases upon pathogen infection [3]. In this plant, SA biosynthesis begins with chorismate localized in the chloroplast and occurs via a relatively simple pathway involving only two intermediate compounds: isochorismate and isochorismate-9-glutamate [4,5]. The pathway in rice (*Oryza sativa*) is considerably more complex, involving the sequential production of multiple intermediates including phenylalanine, *trans*-cinnamic acid, cinnamoyl-CoA, benzoylacetyl-CoA, benzyl benzoate, and salicyl benzoate [[6], [7], [8], [9]].

Due to the causal relationship between SA and SAR, there is great interest in observing the initial SA burst within the pathogen infection site as well as the transmission of SA in living tissue. Yet, the most common method for SA analysis, mass spectrometry, is inherently static due to destructive sampling. This method can only provide a “snapshot” of a specific spatiotemporal point instead of capturing the real-time dynamic changes in SA levels within the plant. This limitation hinders our ability to analyze the spatiotemporal dynamics of SA signaling during early immune responses and to uncover the sequential interactions between SA and early signals such as calcium ions and reactive oxygen species (ROS). Consequently, the development of imaging techniques capable of monitoring SA dynamics *in vivo*, in real time, at the site of action has become a critical goal in order to gain deeper insights into the mechanisms underlying SAR.

In a recent article entitled “SALICYLIC ACID SENSOR1 reveals the propagation of an SA hormone surge during plant pathogen advance” published in *Science*, Tang et al. reveal the spatiotemporal dynamics of pathogen-induced SA biosynthesis in plants [10]. The authors successfully developed a genetically encoded fluorescent SA sensor, SalicS1, which functions like a “real-time camera”, enabling the live visualization and tracking of SA dynamics in intact plants. This work revealed a previously unknown phenomenon: during pathogen infection, a rapid wave of SA accumulation propagates ahead of the invading pathogen through as-yet-uninfected cells. This discovery directly captures a “forward wave” of plant defense responses and provides key real-time observations that could help decipher the signaling mechanism underlying SAR. These findings clearly demonstrate that plants do not passively await invasion but actively and systematically propagate danger signals across different cells.

The design of SalicS1 was inspired by the SA-induced disruption of the interaction between the plant's natural SA receptor Nonexpresser of Pathogenesis-Related genes 1 (NPR1) and NIMIN proteins [11]. Rather than using full-length NPR1 protein, only the specific domain involved in SA recognition and binding was employed as the sensing core. This strategy successfully reduced the sensor's molecular size, thereby enhancing its expression and diffusion within cells while avoiding interference with the native NPR1 signaling pathway and minimizing potential impacts on plant physiology. SalicS1 adheres to the standard architecture of a genetically encoded FRET biosensor, comprising a donor fluorescent protein (FP), an SA-binding domain, and an acceptor FP (Fig. 1). A key refinement involves the use of a circular permutation of FP, which was created by “cutting and rejoining” the amino acid sequence to enhance its sensitivity to conformational dynamics. In its SA-free state, NtNPR1t and NtNIMIN1 interact, bringing the donor and acceptor FPs into closer physical proximity, thereby enabling Donor-excited Acceptor emission (Fig. 1). SA induction triggers conformational changes to the interacting proteins, thereby disrupting their interaction. This allosteric effect is subsequently transmitted to the FPs, leading to a measurable decrease in the emission ratio (Donor-excited Acceptor emission/Donor-excited Donor emission) (Fig. 1). The extent of change to the emission ratio is directly proportional to SA concentration, as increased occupancy of the binding domains leads to more pronounced conformational changes. Consequently, SalicS1 operates as a sensor whose emission ratio is inversely proportional to SA concentration (Fig. 1). In *in vitro* experiments, after washing out exogenous SA with buffer, the sensor signal in plants returned to its pre-treatment level, demonstrating the sensor's capability for repeated detection.

**Fig. 1:**
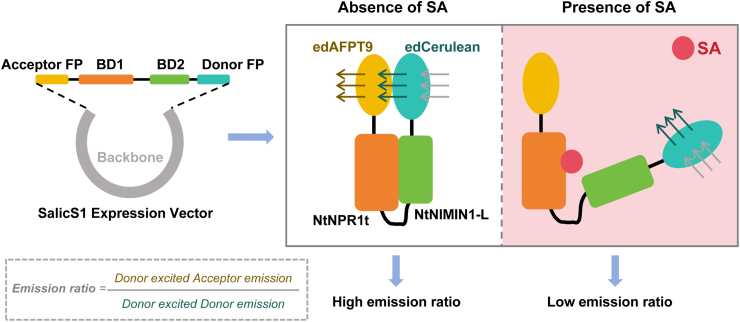
Vector construction and working model of the SalicS1 sensor. The expression vector for the SalicS1 sensor contains four principal functional domains, encoding the acceptor fluorescent protein edAFP19, the donor fluorescent protein edCerulean, and a pair of interacting proteins, NtNPR1t and NtNIMIN1-L. When little or no SA is present, a pronounced FRET effect occurs between the donor and acceptor fluorescent proteins, resulting in a high emission ratio. By contrast, in the presence of sufficient levels of SA, the interaction between NtNPR1t and NtNIMIN1-L is disrupted, leading to attenuated FRET signals and a reduced emission ratio.

Compared to conventional gene expression reporting systems like PR1GFP, the SalicS1 system directly detects the concentration of the phytohormone itself, rather than downstream gene expression. This direct measurement offers greater responsiveness and avoids interference from delayed signal transduction. The propagation process of the SA “wave” identified in this study is noteworthy: rather than uniform diffusion of SA, the authors observed an “SA wave” advancing at a specific velocity. The observed wave velocity was dramatically slower than would be expected for simple diffusion of a small molecule. The front of this wave progressed in synchrony with the expansion of the pathogen. The formation of this wave depended not only on successful infection but on also the spread of the pathogen. Thus, in *Arabidopsis*, only the host-adapted pathogenic bacterium *Pst* DC3000 but not the non-host-adapted fungus *Blumeria graminis* or the aphid *Brevicoryne brassicae* induced such an SA wave.

How does an SA burst at the infection site trigger an SA wave in neighboring cells? This process likely involves a signal relay mechanism. First, the initial burst of SA at the infection site induces the biosynthesis of key mobile signal precursors, particularly pipecolic acid (Pip). Subsequently, Pip is converted into its biologically active derivative, N-hydroxypipecolic acid (NHP). Second, these mobile signals, primarily NHP and potentially Pip itself, move to neighboring cells [12,13]. Upon their arrival, these signals activate the cell's intrinsic SA biosynthetic pathway, thereby propagating a secondary wave of SA production and establishing a feed-forward amplification loop for systemic immunity.

To investigate how SA coordinates with other early signaling molecules and to delineate its leading role in temporal and spatial gradients, high-resolution live imaging based on genetically encoded fluorescent indicators (e.g., SA: SalicS1 sensor; ROS: HyPer; Ca^2+^: GCaMP6) could be employed [10,14,15]. However, when multiplexing these indicators, potential spectral interference between their respective excitation and emission wavelengths must be carefully considered. For instance, GCaMP6 exhibits excitation/emission at 488/510 nm, while the YFP component of the SalicS1 FRET pair shows excitation/emission at 500/530 nm. The close proximity of these spectra indicates significant overlap in both excitation (488 nm vs. 500 nm) and emission (510 nm vs. 530 nm) channels. Similarly, HyPer, with an emission peak at 535 nm, falls within the same emission range as GCaMP6 and YFP, leading to potential bleed-through. Additionally, its 500 nm excitation peak overlaps with that of YFP [10,14,15]. This substantial spectral crosstalk could compromise signal specificity.

To mitigate this issue, the development of indicators with minimal spectral overlap should be prioritized. In this context, recently developed ROS sensors such as roGFP2-Orp1 and roGFP2-PRXIIB offer distinct advantages [16,17]. Unlike HyPer, these probes are ratiometric and based on the roGFP2 framework, which exhibits distinct excitation peaks at 405 nm (oxidized) and 488 nm (reduced), with a fixed emission wavelength of 510 nm. This spectral signature, particularly the utilization of the UV/violet (405 nm) excitation channel, reduces spectral crosstalk with commonly used green and yellow fluorescent proteins (e.g., GCaMP6, YFP), thereby facilitating more reliable multiplex imaging. When overlap is unavoidable, sequential scanning (rather than simultaneous acquisition) or spectral unmixing techniques must be employed to ensure signal specificity and data accuracy. By performing multicolor synchronous imaging at localized pathogen infection sites, the relative propagation speeds, effective ranges, and dynamic temporal sequences of SA, ROS, and Ca^2+^ signals could be simultaneously monitored, thereby enabling precise identification of the true “pioneer signal”. This approach holds promise for resolving a long-standing scientific debate: whether SA accumulation acts as the cause or the consequence of ROS/Ca^2+^ bursts.

This approach could be combined with the use of specific mutants or inhibitors that separately block SA biosynthesis, ROS bursts, or calcium channel activity to determine their upstream-downstream regulatory relationships. Moreover, sensor-based platforms could be combined with large-scale genetic screening of mutant libraries to establish automated imaging systems, enabling the screening of mutants with abnormal timing, intensity, propagation range, or spatial localization of SA signaling. The SalicS1 reporter line, in particular, presents a valuable opportunity for such forward genetic screening to identify novel components of SA signaling and propagation. However, when performing these types of screens, it is important to note that isolated mutants may not only affect SA signaling but could also non-specifically interfere with SalicS1 expression, protein stability, or chromophore maturation. Therefore, secondary validation assays (e.g., independent SA quantification by LC-MS/MS, or validation using an alternative SA reporter) are necessary to confirm that the identified mutants specifically affect SA biology rather than reporter function. This approach allows for the direct identification of novel genes involved in regulating SA biosynthesis, metabolism, perception, or transport, as well as the initiation of other immune signaling waves. Beyond genetic screening, by introducing the SA sensor into diverse crop varieties or local germplasms and comparing the initiation speed, peak intensity, and persistence of SA signals upon infection, a quantitative resistance evaluation system of crops based on early defense signaling response could be established, potentially facilitating breeding.

Notably, SalicS1 employs a nuclear localization sequence (NLS) to enhance the visualization of SA in the nucleus, a position most relevant to the signaling function of SA. Yet, it is still unclear whether or how much SA accumulates in different subcellular compartments, which is intriguing considering the high basal SA levels in different species and organs. The further evolution of SalicS1, including the development of compartment-specific versions of this sensor, may unleash greater potential. Nevertheless, the invention of SalicS1 opened up the new research field of “real time spatiotemporal dynamics of plant SA and immune signaling”, which is expected to have broad, profound theoretical and technical impacts on the study of plant immunity.

## CRediT authorship contribution statement

**Shanshan Li:** Writing – original draft, Formal analysis, Data curation, Conceptualization. **Honghong Cao:** Writing – original draft. **Qiuyue Zhang:** Writing – original draft. **Yukang Wang:** Writing – original draft. **Shancen Zhao:** Writing – original draft. **Ronghui Pan:** Writing – review & editing, Funding acquisition, Formal analysis, Data curation, Conceptualization.

## Declaration of competing interest

The authors declare that they have no known competing financial interests or personal relationships that could have appeared to influence the work reported in this paper.

## Data Availability

No data was used for the research described in the article.
